# Multiple Host–Guest Interactions in Metal–Organic Frameworks Constructed by Inverted Calix[4]arenes

**DOI:** 10.1021/jacs.5c08164

**Published:** 2025-07-14

**Authors:** Zongsu Han, Kun-Yu Wang, Yifan Guo, Ze-Han Wang, Rong-Ran Liang, Yihao Yang, Jiatong Huo, Dong-Sheng Guo, Hong-Cai Zhou

**Affiliations:** † Department of Chemistry, 14736 Texas A&M University, College Station, Texas 77843, United States; ‡ College of Chemistry, Key Laboratory of Functional Polymer Materials (Ministry of Education), State Key Laboratory of Elemento-Organic Chemistry, 12538 Nankai University, Tianjin, 300071, China; # School of Pharmaceutical Science and Technology, Tianjin University, Tianjin 300072, China

## Abstract

Calixarenes are versatile macrocyclic compounds featuring unique basket-like cavities that are capable of encapsulating guest molecules selectively. Yet, their application potentials as the building blocks for supramolecular assemblies have not been thoroughly explored. In this work, a carboxyl-modified azocalix[4]­arene (CAC4A) is selected as the organic ligand to construct metal–organic frameworks (MOFs), an emerging class of porous materials based on coordination units. Herein, three calixarene-based MOFs are developed based on varied metals, including La^3+^, Ca^2+^, and Mn^2+^. Benefiting from the low symmetry of the calixarene ligands, the three MOFs feature abundant structural diversity, wherein two inverted calixarenes are bridged by metal nodes to produce nanocavities. These cavities, or pores, are interconnected to generate one-dimensional (1D) chains or two-dimensional (2D) sheets that are further assembled into porous frameworks. Such a bottom-up assembly not only presents an approach to constructing hierarchical porous structures, but also gives rise to enhanced adsorption abilities based on host–guest interactions. Single crystal X-ray diffraction can also be employed to determine the interactions between the guest molecule, like iodine, and the frameworks directly.

## Graphical abstract

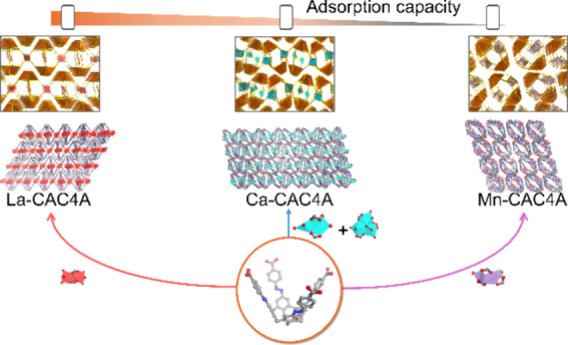

Metal–organic frameworks (MOFs) are an emerging class of porous materials consisting of metal ions and organic ligands,^1–3^ which have garnered significant interest in various applications, including guest adsorption,^4–7^ heterogeneous catalysis,^8–11^ and molecule recognition.^12–15^ Their chemical structures can be meticulously designed and engineered to achieve tailored pore sizes, shapes, and functionalities.^16–20^ One cornerstone in developing functional MOFs is by incorporating specific binding sites for guest molecules. Calixarenes are significant macrocyclic compounds that comprise phenolic units linked by methylene, forming cavities capable of encapsulating various guest molecules.^21–23^ Such a structural feature brings about engaging properties and application potentials in sensing,^24,25^ pollutant removal,^26,27^ and drug delivery.^28,29^ In particular, introducing calixarenes into MOF structures will endow the materials with hierarchical pore environments, where the nanosized cavities of calixarenes can be interconnected periodically, generating permanent pores or channels to accommodate guest molecules. However, due to the synthetic difficulties, current reports on macrocyclic-ligand-based MOFs are still rare,^30–33^ and calixarenes often serve as the capping ligands or guest molecules in MOFs merely.^34–37^ In addition, mechanism studies on the interactions between calixarene-based MOFs and guest molecules are not sufficient.

In this work, a carboxyl-modified azocalix[4]­arene, named 5,11,17,23-tetrakis­[(*p*-carboxyphenyl)­azo]-25,26,27,28-tetra-hydroxy calix[4]­arene (CAC4A), is selected as the ligand to construct a series of calixarene-based MOFs, namely La-CAC4A, Mn-CAC4A, and Ca-CAC4A. Intriguingly, it is found that two inverted calixarenes are prone to forming a pocket with a size of 2.3 nm × 1.2 nm, as connected by metal nodes in these MOFs, which serves as the primary building block to generate 1D chains or 2D sheets. The two conformationally inverted calix[4]­arene molecules act as a building block coordinated with diverse metal centers, enabling the formation of a variety of MOF structures. Its preserved configurations observed in different assemblies highlight its potential as a universal module for the construction of hierarchical supramolecular assemblies. Herein, La-CAC4A features the pores of both calixarenes and frameworks, possessing higher adsorption capacities compared with Mn-CAC4A, Ca-CAC4A, and some classical MOFs, which can be attributed to the binding affinity as well as tailored molecular configurations of calixarenes.

La-CAC4A, Mn-CAC4A, and Ca-CAC4A were synthesized using the solvothermal method. According to the results of single crystal X-ray diffraction (SCXRD) (Table S1), the minimum asymmetric unit of La-CAC4A consists of two La ions, one *N*,*N*-dimethylformamide (DMF), seven water, and one calixarene. The La ions are 9-coordinated, which are connected by four COO^–^ to form La_2_(DMF)_2_(H_2_O)_6_(COO)_4_/La_2_(H_2_O)_8_(COO)_4_ nodes, while each {La_2_} node is linked with four calixarenes to afford a 2D (4,4)-connected sheets with **sql** topology (Figure 1a). Interestingly, the nanosized pockets in La-CAC4A are spaced by the {La_2_} nodes, generating 1.25 nm × 0.96 nm parallelogram pores. For Mn-CAC4A, the minimum asymmetric unit of Mn-CAC4A consists of three Mn ions, six DMF, one HCOO^–^, and one calixarene ligand, while two Mn ions are 6-coordinated and one Mn ion is 5-coordinated. The three Mn ions are connected by one HCOO^–^ and four COO^–^ to form a Mn_3_(DMF)_6_(HCOO)­(COO)_4_ cluster, which is further linked with four calixarenes to afford a 1D (4,4)-connected chain (Figure 1b). The Mn-CAC4A framework is constructed through the parallel alignment of the 1D chains, wherein the dense packing mode may hinder the accessibility of the nanosized pocket between two calixarenes. As a result, a narrow window with a size of 1.60 nm × 0.45 nm is exposed between the two inverted calix[4]­arenes in Mn-CAC4A. In addition, for Ca-CAC4A, there are five Ca ions, four DMF, nine water, one μ_2_-O, and two calixarenes in the minimum asymmetric unit, while four Ca ions are 8-coordinated and one Ca ion is 7-coordinated. It should be noted that there are two types of metal clusters in Ca-CAC4A, including Ca_3_(DMF)­(H_2_O)_5_(COO)_6_ and Ca_2_(μ_2_-O)­(DMF)_3_(H_2_O)_4_(COO)_2_. Each {Ca_3_} node and {Ca_2_} node are linked with six calixarenes and two calixarenes, respectively, yielding a 2D (2,4,6)-connected sheet featuring a 0.36 nm × 2.20 nm window (Figure 1c). The sheets are densely packed through AA stacking to form the Ca-CAC4A framework, wherein the nanosized pockets between calixarenes are staggered.

**Figure 1:**
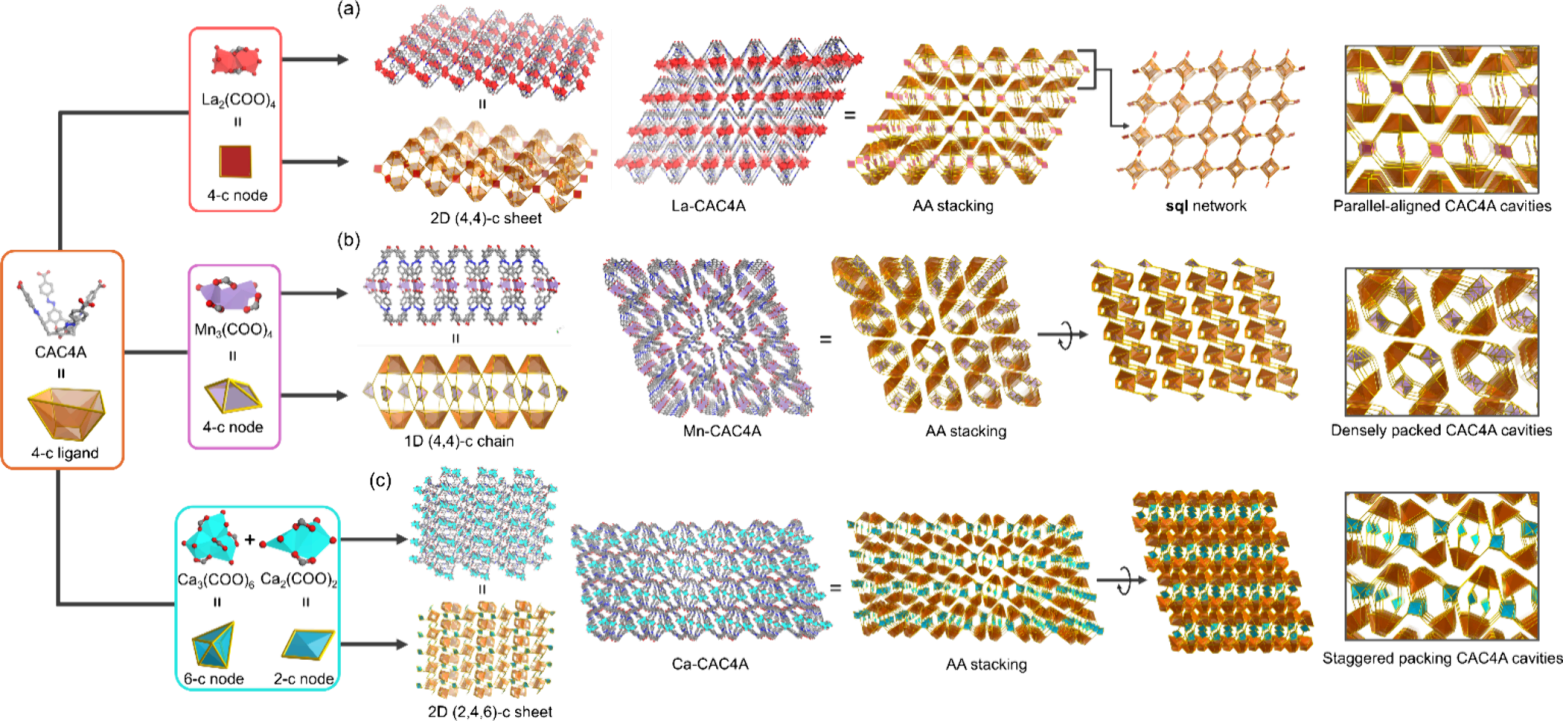
Construction of La-CAC4A, Mn-CAC4A, and Ca-CAC4A based on the 4-connected CAC4A ligand. (a) Structural illustration of the La-CAC4A based on stacking 2D (4,4)-connected sheets, which consist of 4-connected {La_2_} clusters and CAC4A ligands. (b) Structural illustration of the Mn-CAC4A based on stacking 1D (4,4)-connected chains, which comprise 4-connected {Mn_3_} clusters and CAC4A ligands. (c) Structural illustration of the Ca-CAC4A based on the stacking of 2D (2,4,6)-connected sheets, which consist of 6-connected {Ca_3_} clusters, 2-connected {Ca_2_} clusters, and CAC4A ligands.

Powder X-ray diffraction (PXRD) patterns confirm the high crystallinity and phase purity of these MOFs (Figure S1). Thermogravimetric analysis (TGA) of these materials indicates continuous mass loss due to the presence of solvent molecules in their pores (Figure S2). Images under the optical microscope (Figure S3) and scanning electron microscope (SEM) (Figure S4) show that La-CAC4A, Mn-CAC4A, and Ca-CAC4A are in block, rod, and block shapes, respectively. When comparing the ultraviolet–visible (UV–vis) spectroscopy^38^ of these MOFs with the ligand, the UV–vis absorption peaks are widened, while the peaks feature red shifts (Figure S5), confirming the coordination structures.

According to the crystallographic structures determined by SCXRD, the porosity of these MOFs is different. Herein, iodine is selected to study how their pore environments affect adsorption performances, because of its appropriate molecular sizes and feasibility to be identified using SCXRD, making it an ideal probe for evaluating host–guest interactions, porosity, and adsorption mechanisms in porous materials. In a typical iodine adsorption experiment, 5 mg MOF crystals are added into 3 mL iodine with a concentration of 10 μg mL^–1^ in DMF. Based on the UV–vis intensity changes (Figure S6), La-CAC4A exhibits much faster iodine adsorption performance than Ca-CAC4A and Mn-CAC4A, benefiting from its abundant pores consisting of both the calixarene cavities and the MOF pores. Ca-CAC4A shows slightly faster iodine adsorption than Mn-CAC4A, as the formation of 1D chains in Mn-CAC4A may hinder the ingress of guest molecules (Figure 2a). As shown in Figure 2b, the adsorption processes exhibit fast kinetics, which gives adsorption rate constants (*k*_2_) for La-CAC4A, Ca-CAC4A, and Mn-CAC4A as 5.62 × 10^–1^, 1.82 × 10^–3^, and 1.49 × 10^–3^ g mg^–1^ h^–1^, respectively, according to the pseudo-second-order kinetics model.^39^

**Figure 2:**
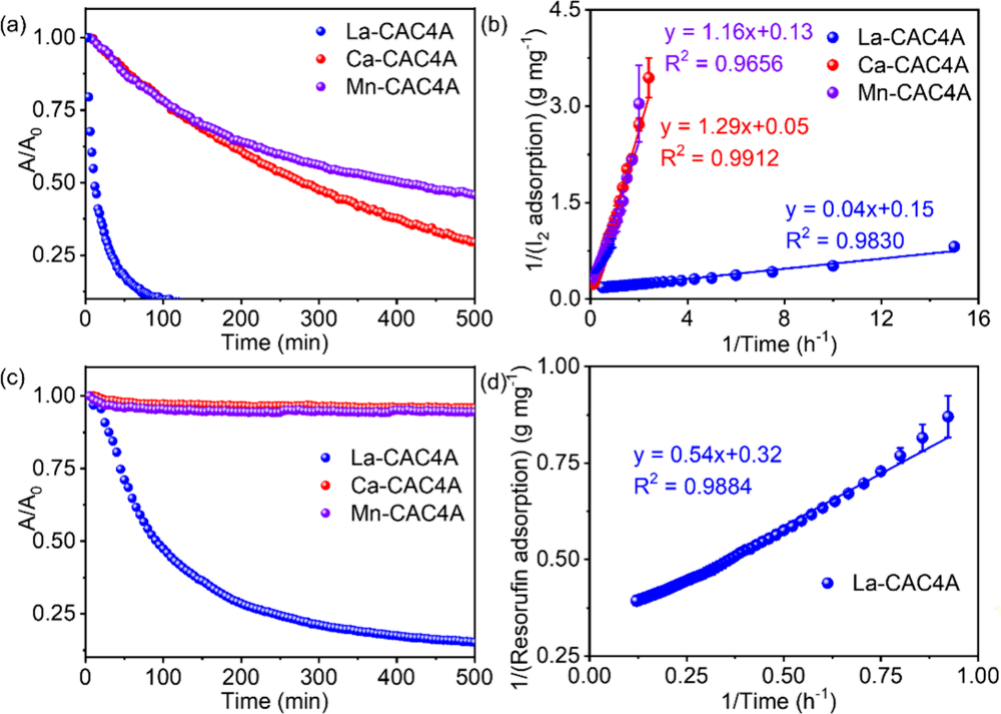
UV–vis intensity changes (a) and sorption kinetics (b) of I_2_ with the time after the additions of MOFs. UV–vis intensity changes (c) and sorption kinetics (d) of resorufin with the time after the additions of MOFs.

To further confirm the porosity, a larger molecule, resorufin, with a size of 1.03 nm × 0.51 nm is selected to evaluate their pore sizes, and 5 mg MOF crystals are added into 5 μg mL^–1^ resorufin in 3 mL of DMF. Based on the UV–vis intensity changes (Figure S7), La-CAC4A can effectively adsorb resorufin, while no apparent adsorption is observed in Ca-CAC4A and Mn-CAC4A (Figure 2c). Such a difference highlights the larger pore size of La-CAC4A compared with Ca-CAC4A and Mn-CAC4A, further confirming the presence of hierarchical pores in La-CAC4A. Besides, as shown in Figure 2d, the *k*_2_ value for La-CAC4A toward resorufin is 1.85 × 10^–1^ g mg^–1^ h^–1^ based on the pseudo-second-order kinetics model.^39^ The adsorption of resorufin in La-CAC4A can be further confirmed by ^1^H nuclear magnetic resonance (NMR) spectroscopy, which gives the ratio between resorufin and calixarene as 1:4 (Figure S8).

Furthermore, the iodine adsorption performance of La-CAC4A is compared with a series of classical MOFs, including CALF-20,^40^ CAU-1,^41^ CAU-3-NH_2_,^42^ CAU-4,^43^ HKUST-1,^44^ MFM-300-In,^45^ MOF-76-Gd,^46^ MOF-177,^44^ UiO-66,^47^ UiO-67,^48^ and ZIF-8 ^49^ (Figures S9–S19), wherein La-CAC4A exhibits the fastest adsorption toward iodine compared with other MOFs (Figure 3), indicating the superiority of the hierarchical pore structures in sorption.

**Figure 3:**
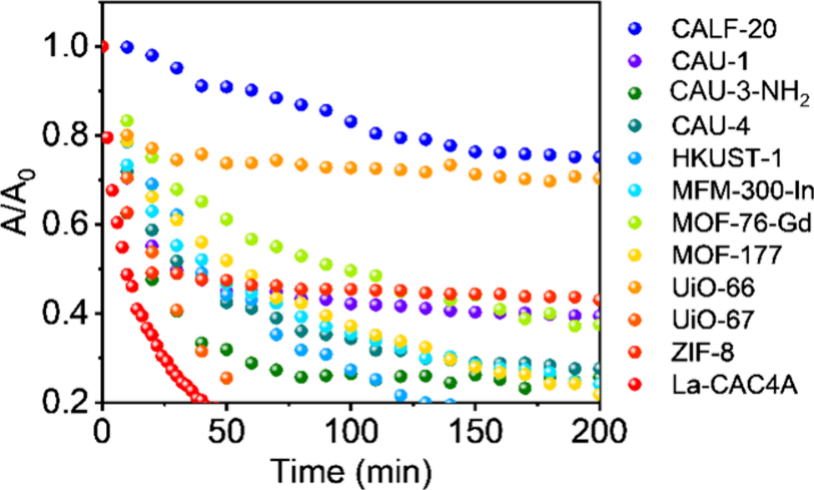
Comparison of the iodine adsorption efficiencies of La-CAC4A with some classical MOFs.

The binding sites of iodine in these MOFs are analyzed by SCXRD (Figure 4). MOFs are soaked in DMF solutions of iodine for 7 days, then the positions of the iodine can be solved through the diffraction patterns. Multiple interactions are observed in La-CAC4A to bind iodine molecules (Figure 4a). One iodine molecule can connect with the calixarene through π···π interaction at 4.1 Å, while the other iodine molecule is synergistically bound by a calixarene ligand and a water molecule through three hydrogen bonds of 2.5, 2.8, and 2.8 Å, respectively. There are two main positions observed in Mn-CAC4A to accommodate iodine molecules. The first one is connected to the DMF molecule coordinated on the metal center through three hydrogen bonds at lengths of 2.5, 2.6, and 2.7 Å, respectively. The second position is connected to the calixarene through one 2.7 Å hydrogen bond. (Figure 4b) In Ca-CAC4A, iodine molecules can bond with the calixarene ligand through π···π interactions at 4.0 Å and 3.8 Å (Figure 4c). In addition, iodine can be merely found inside the calixarene cavities in Mn-CAC4A and Ca-CAC4A, while it can occupy both inner and outer pores of the calixarene in La-CAC4A. To further investigate the host–guest interactions between the iodine and the calix[4]­arene ligand, density functional theory (DFT) calculations are conducted. Using the B2-PLYP functional and D3BJ dispersion correlation the results further support the experimental findings (Figure S20). The single-point energy calculations of optimized structures indicate that the iodine’s binding with an azobenzene monomer is endothermic, suggesting an unfavorable thermodynamic driving force under standard conditions. While iodine’s encapsulation within the CAC4A’s nanocavity is an exothermic process, confirming the calix[4]­arene’s advantage in selectively accommodating the guest molecules.

**Figure 4:**
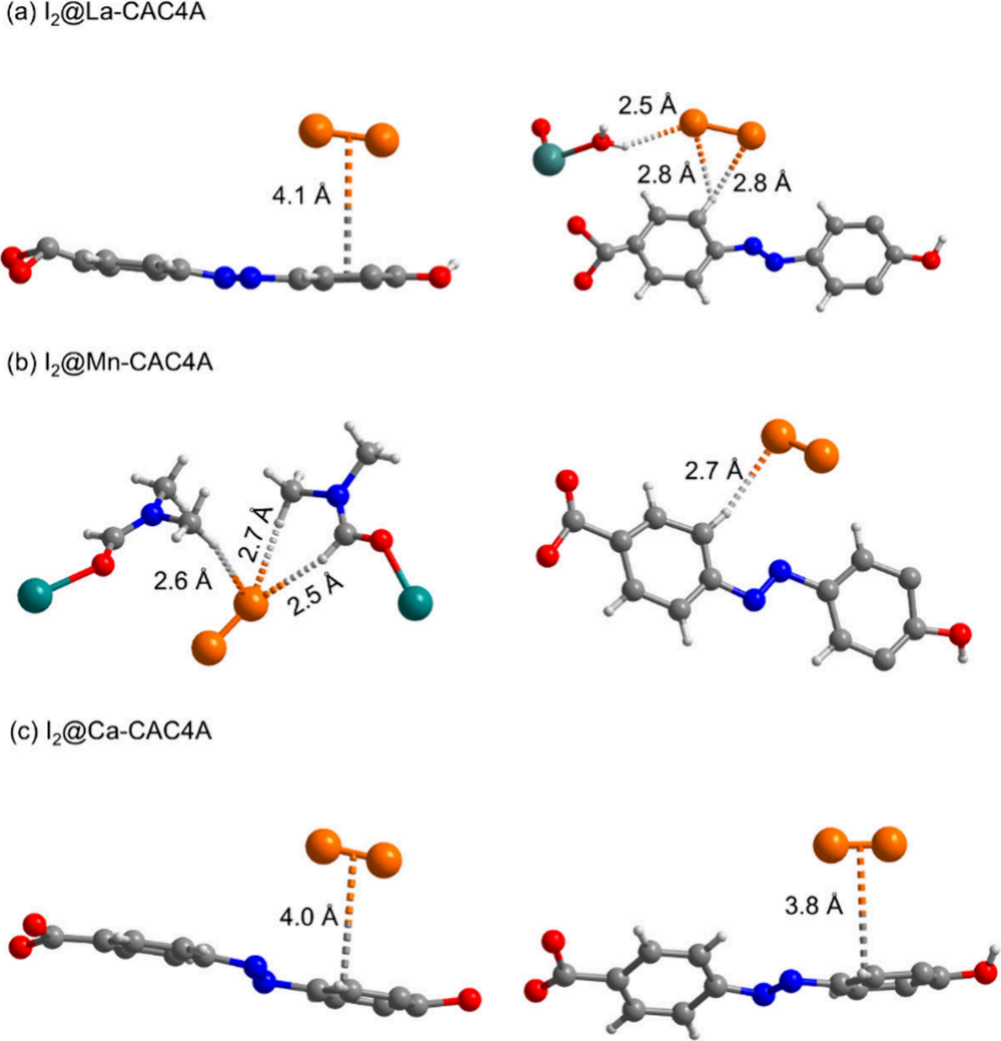
Binding sites of iodine in La-CAC4A (a), Mn-CAC4A (b), and Ca-CAC4A (c) from SCXRD data. Atom code: C, gray; N, blue; O, red; I, orange; La/Mn, teal.

In summary, three different MOFs constructed with the same calix[4]­arene ligand are successfully synthesized and characterized, where two inverted calixarene molecules are interconnected to afford nanosized cavities and further assembled into low-dimensional chains or sheets. Such an inverted conformation of the two calix[4]­arene-based ligands tends to form spontaneously under the synthetic conditions. Most importantly, such a structure serves as a versatile building block, which can be further linked by different metal nodes to construct MOFs with various topologies. The structural consistency of the inverted calixarene pocket widely observed in different MOFs suggests that it can act as a structural module for assembling materials with diverse pore environments.

Among these MOFs, La-CAC4A features both the calixarene cavity and the MOF pore, endowing the material with the superior iodine adsorption ability even compared with some classical MOFs. The binding sites for iodine in these MOFs can also be resolved by SCXRD, which confirms multiple interactions to accommodate iodine in these calixarene-based MOFs. This work presents a bottom-up approach to diversifying chemical structures and pore environments of MOFs, leveraging the intrinsic cavities and host–guest interactions of supramolecules, which will pave the pathway to develop supramolecular materials for efficient adsorption and recognition in heterogeneous systems.

## Supplementary Material



## Notes

Materials and methods, basic characterizations, adsorption abilities, and relative tables. (PDF)
