# Supplementary material for: Multiple Host–Guest Interactions in Metal–Organic Frameworks Constructed by Inverted Calix[4]arenes
Source: J Am Chem Soc. 2025 Jul 14;147(29):25162–6. doi: 10.1021/jacs.5c08164 (PMC12291452; doi:10.1021/jacs.5c08164)
Supplement: Supplementary file 1 [file ja5c08164_si_001.pdf]

Supporting Information

Multiple Host-Guest Interactions in Metal-Organic Frameworks Constructed by Inverted Calix[4]arenes

Zongsu Han,[†,§] Kun-Yu Wang,[†,§] Yifan Guo,[#] Ze-Han Wang,[‡] Rong-Ran Liang,[†] Yihao Yang,[†] Jiatong Huo,[†] Dong-Sheng Guo,[‡,]* and Hong-Cai Zhou[†,]*

[†]Department of Chemistry, Texas A&M University, College Station, Texas 77843, United States
[‡]College of Chemistry, Key Laboratory of Functional Polymer Materials (Ministry of Education), State Key Laboratory of Elemento-Organic Chemistry, Nankai University, Tianjin, 300071 China
[#]School of Pharmaceutical Science and Technology, Tianjin University, Tianjin 300072, China
Correspondence and requests for materials should be addressed to D.-S. G. (dshguo@nankai.edu.cn), H.-C. Z. (email: zhou@chem.tamu.edu)

Methods

Materials and Methods

All reagents were commercially available and used without further purification. Liquid ^{1}H NMR spectra were recorded on a Bruker Avance NEO 400 NMR spectrometer. Single crystal X-ray diffraction patterns were collected by a Bruker-Axs Venture Ius Cmos Kappa X-ray Apex2 diffractometer with Cu-Kα radiation and a Bruker-Axs Quest Ius Three-Circle X-ray Apex2 diffractometer with Mo-Kα radiation. The structures were solved by SHELXS (direct methods) and refined by SHELXL (full matrix least-squares techniques) in the Olex2 package.[1,2] PXRD measurements were performed using a Bruker Powder-ECO X-ray diffractometer with Cu-Kα radiation. Thermo-gravimetric analysis curves were obtained under nitrogen atmosphere on a Mettler Toledo TGA/DSC 1 thermogravimetric analyzer from 40 °C to 800 °C. UV-vis absorption spectra were measured by a Shimadzu UV-2450 Spectrometer. SEM was carried out using a FEI QUANTA 600 FE-SEM scanning electron microscope. 3 mg MOFs for NMR tests were dissolved by 0.5 mL d_6-DMSO and 5 μL D_2SO_4.

Synthesis

Synthesis of La-CAC4A.

50 mg $La(NO_3)_3·6H_2O$ and 10 mg CAC4A were dissolved in 2 mL DMF. The solution was placed in a 20 mL glass bottle and heated at 120 °C for one day. Finally, the samples were separated by a centrifugation, washed with fresh DMF for three times, and dried in air.

Synthesis of Mn-CAC4A.

50 mg $MnCl_2$ and 10 mg CAC4A were dissolved in 2 mL DMF. The solution was placed in a 20 mL glass bottle and heated at 120 °C for one day. Finally, the samples were separated by a centrifugation, washed with fresh DMF for three times, and dried in air.

Synthesis of Ca-CAC4A.

50 mg $Ca(NO_3)_2·4H_2O$ and 10 mg CAC4A were dissolved in 2 mL DMF. The solution was placed in a 20 mL glass bottle and heated at 120 °C for one day. Finally, the samples were separated by a centrifugation, washed with fresh DMF for three times, and dried in air.

Synthesis of CALF-20.

CALF-20 is composed of layers of 1,2,4-triazolate-bridged Zinc(II) ions pillared by oxalate ions to form a 3D lattice and 3D pore structure.[3]

6.6 g Zinc oxalate and 5 g 1,2,4-triazole were dissolved in 66 mL methanol. The solution was placed in a 100 mL Teflon-sealed autoclave and heated at 180 °C for one day. Finally, the samples were separated by a centrifugation, washed with fresh methanol for three times, and dried in air.

Synthesis of CAU-1.

CAU-1 is built up from a pseudobody-centered-cubic arrangement of the 8-ring building blocks. The resulting 3D microporous framework involves distorted octahedral and tetrahedral cages.[4]

1 g $AlCl_3$ and 250 mg 2-aminoterephthalic acid were dissolved in 10 mL methanol. The solution was placed in a 23 mL Teflon-sealed autoclave and heated at 120 °C for one day. Finally, the samples were separated by a centrifugation, washed with fresh methanol for three times, and dried in air.

Synthesis of CAU-3-NH₂.

The structure of CAU-3-BDC-NH_2 is based on dodecameric cations. The resulting network contains tetrahedral and octahedral cavities.[5]

270 mg $Al(NO_3)_3 \cdot 9H_2O$, 20 mg 2-aminoterephthalic acid and 20 mg NaOH were dissolved in 8 mL methanol. The solution was placed in a 23 mL Teflon-sealed autoclave and heated at 120 °C for one day. Finally, the samples were separated by a centrifugation, washed with fresh methanol for three times, and dried in air.

Synthesis of CAU-4.

The framework structure of CAU-4 consists of isolated [AlO_6]-octahedra which are bridged by carboxylate groups of the linker to form chains. These chains are connected by the aromatic building blocks, to form 1D hexagonal channels.[6]

51 mg $Al(NO_3)_3 \cdot 9H_2O$, 30 mg benzoic acid and 120 mg 1,3,5-benzenetrisbenzoic acid were dissolved in 1.5 mL DMF. The solution was placed in a 23 mL Teflon-sealed autoclave and heated at 180 °C for one day. Finally, the samples were separated by a centrifugation, washed with fresh DMF and methanol for three times respectively, and dried in air.

Synthesis of HKUST-1.

HKUST-1 is composed of dimeric cupric tetracarboxylate units, with 3D connected network of pores.[7]

438 mg $Cu(NO_3)_2 \cdot 3H_2O$ and 210 mg 1,3,5-benzenetricarboxylic acid were dissolved in 6 mL ethanol and 6 mL water. The solution was placed in a 20 mL glass bottle and heated at 100 °C for one day. Finally, the samples were separated by a centrifugation, washed with fresh ethanol for three times, and dried in air.

Synthesis of MFM-300-In.

MFM-300(In) exhibits an open structure consisting of chains of [$InO_4(OH)_2$] moieties bridged by tetracarboxylate ligands to afford a porous structure with channels formed by corner-sharing octahedra linked via two mutually cis-μ_2-OH groups.[8]

293 mg $In(NO_3)_3$ and 165 mg biphenyl-3,3',5,5'-tetracarboxylic acid were dissolved in 10 mL DMF, 5 mL acetonitrile and 0.5 mL concentrated nitric acid. The solution was placed in a 20 mL glass bottle and heated at 90 °C for one day. Finally, the samples were separated by a centrifugation, washed with fresh DMF and methanol for three times respectively, and dried in air.

Synthesis of MOF-76-Gd.

Each central Gd(III) ion is coordinated by seven oxygen atoms. The [GdO_7] donor set generates polyhedra with pentagonal bipyramidal shape. The porous framework contains 1D channels propagating along the c crystallographic axis.[9]

230 mg $Gd(NO_3)_3 \cdot 6H_2O$ and 53 mg 1,3,5-benzenetricarboxylic acid were dissolved in 4 mL DMF and 4 mL water. The solution was placed in a 20 mL glass bottle and heated at 105 °C for one day. Finally, the samples were separated by a centrifugation, washed with fresh DMF and methanol for three times respectively, and dried in air.

Synthesis of MOF-177.

There are 84 exposed edges (60 C-C, 12 C-O, and 12 Zn-O) and only 4 fused edges (Zn-O) per formula unit. A remarkably open 3D structure of composition MOF-177, in which each basic zinc acetate cluster is linked to six BTB units.[10]

368 mg $Zn(NO_3)_2 \cdot 6H_2O$ and 180 mg 4,4',4''-benzene-1,3,5-triyl-tribenzoic acid were dissolved in 10 mL DEF. The solution was placed in a 20 mL glass bottle and heated at 100 °C for one day. Finally, the samples were separated by a centrifugation, washed with fresh DEF and methanol for three times respectively, and dried in air.

Synthesis of UiO-66.

UiO-66 possesses $Zr_6O_4(OH)_4(CO_2)_{12}$ cluster, which is 12 coordinated by linkers. The structure formed with linear ligands is therefore all expanded versions of the cubic close packed network.[11]

2.5 g $ZrCl_4$ and 2.5 g terephthalic acid were dissolved in 300 mL DMF and 20 mL concentrated hydrochloric acid. The solution was placed in a 500 mL glass bottle and refluxed at 120 °C for one day. Finally, the samples were separated by a centrifugation, washed with fresh DMF and methanol for three times respectively, and dried in air.

Synthesis of UiO-67.

UiO-67 possesses $Zr_6O_4(OH)_4(CO_2)_{12}$ cluster, which is 12 coordinated by linkers. The structure formed with linear ligands is therefore all expanded versions of the cubic close packed network.[11]

125 mg $ZrCl_4$ and 125 mg 4,4'-biphenyldicarboxylic acid were dissolved in 15 mL DMF and 1 mL concentrated hydrochloric acid. The solution was placed in a 23 mL Teflon-sealed autoclave and heated at 120 °C for one day. Finally, the samples were separated by a centrifugation, washed with fresh methanol for three times, and dried in air.

Synthesis of ZIF-8.

ZIF-8 is a zeolitic imidazolate framework composed of Zn^{2+} ions tetrahedrally coordinated by 2-methylimidazolate linkers, forming a sodalite topology.[12]

7.5 g $Zn(NO_3)_2 \cdot 6H_2O$ and 16.5 g 2-methylimidazole were dissolved in 500 mL methanol. Then the solution was placed under ultrasound for six hours. Finally, the samples were separated by a centrifugation, washed with fresh methanol for three times, and dried in air.

DFT Calculation

All quantum chemical calculations were performed using the TURBOMOLE V7.5 program package.[13] Geometry optimizations and energy calculations were carried out using the B2-PLYP double-hybrid density functional[14] in conjunction with Grimme's D3BJ dispersion correction.[15] The def-TZVP basis set[16] was employed for all atoms.

Adsorption Experiments

For all adsorption experiments, 5 mg MOF samples are added into 3 mL iodine DMF solution with a concentration of 10 μg mL^{-1} or 3 mL resorufin DMF solution with a concentration of 5 μg mL^{-1}. Samples for single crystal X-ray diffraction tests were soaked for about one week with exchange of fresh solutions once a day.

Figures

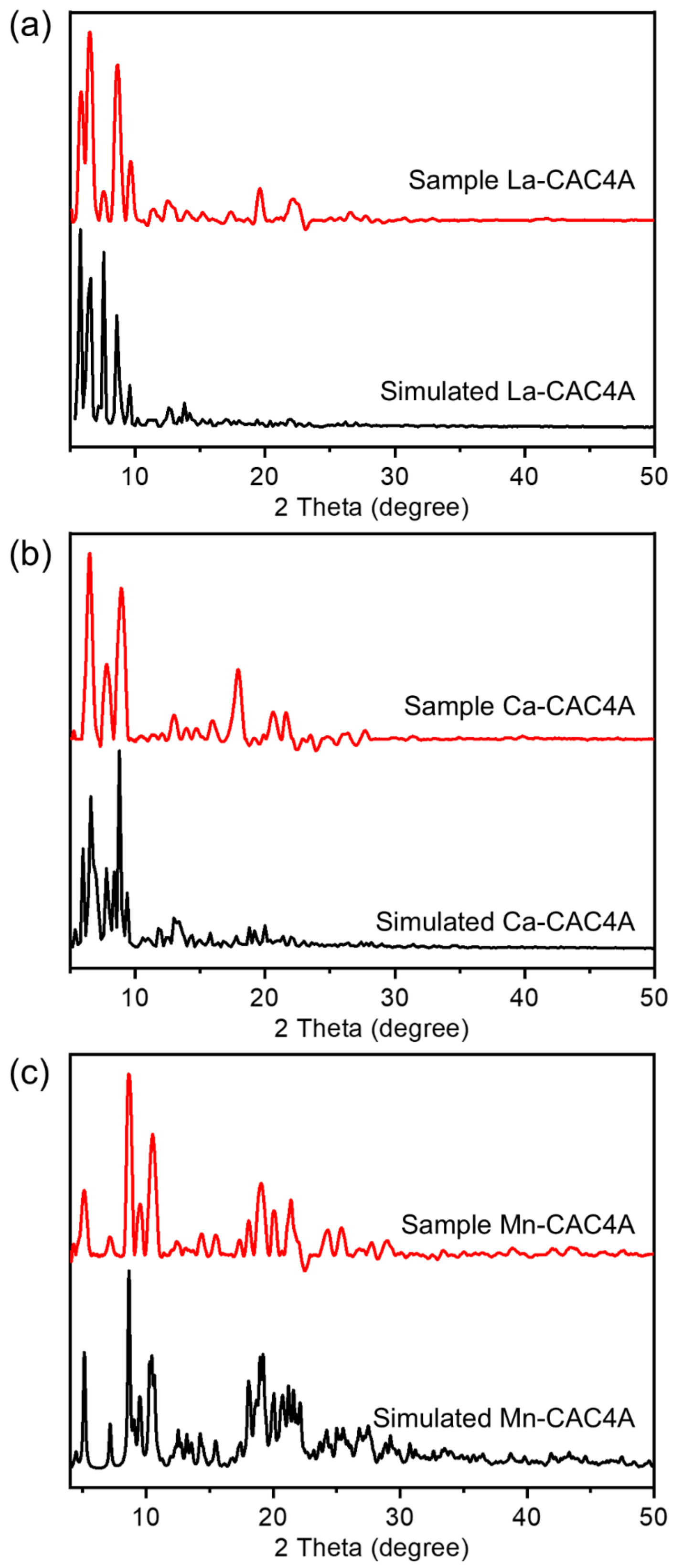

Figure S1. PXRD patterns of calculated and synthesized La-CAC4A (a), Ca-CAC4A (b), and Mn-CAC4A (c).

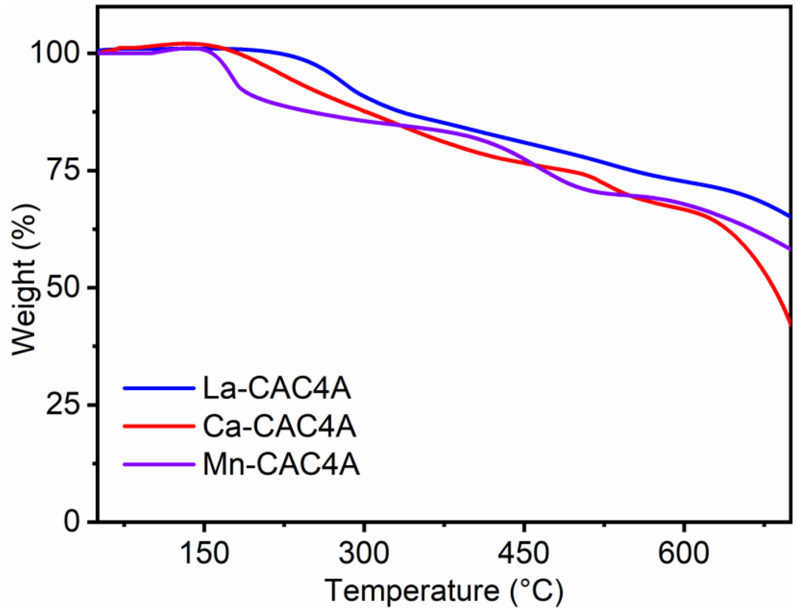

Figure S2. TGA curves of La-CAC4A, Ca-CAC4A, and Mn-CAC4A under nitrogen atmosphere.

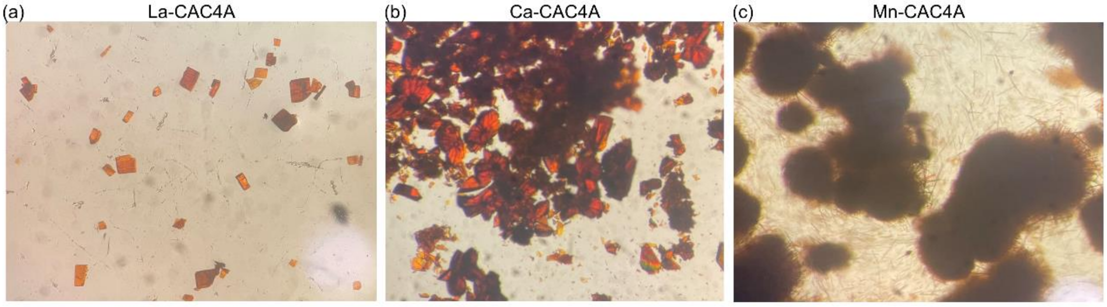

Figure S3. Photos of La-CAC4A (a), Ca-CAC4A (b), and Mn-CAC4A (c) under microscrope.

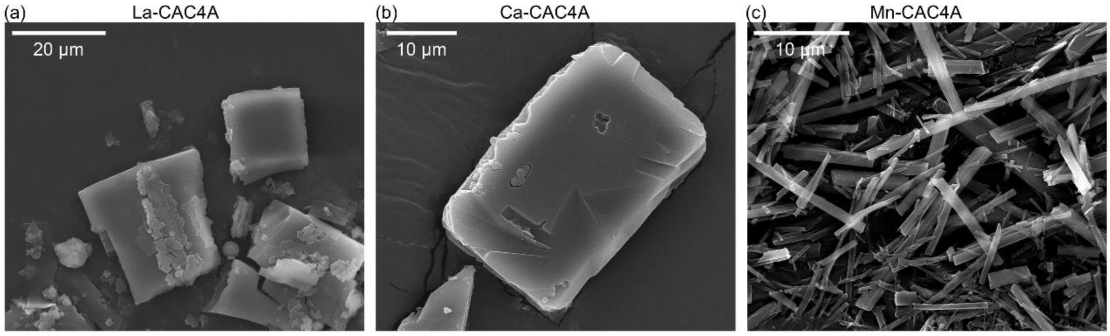

Figure S4. SEM images of La-CAC4A (a), Ca-CAC4A (b), and Mn-CAC4A (c).

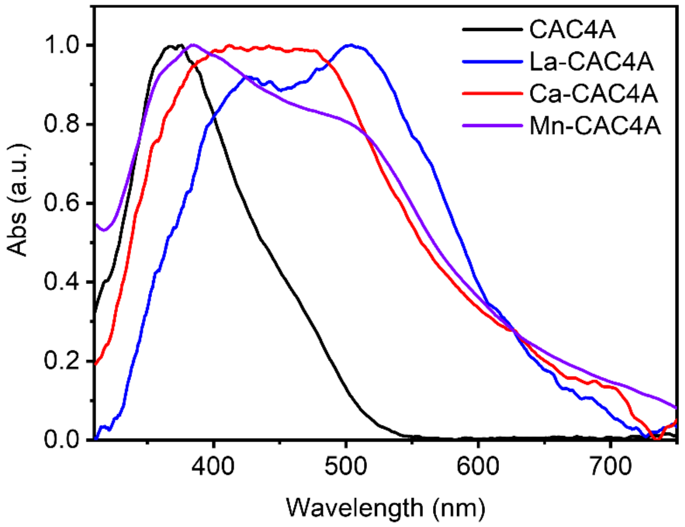

Figure S5. UV-vis spectra of CAC4A, La-CAC4A, Ca-CAC4A, and Mn-CAC4A in DMF.

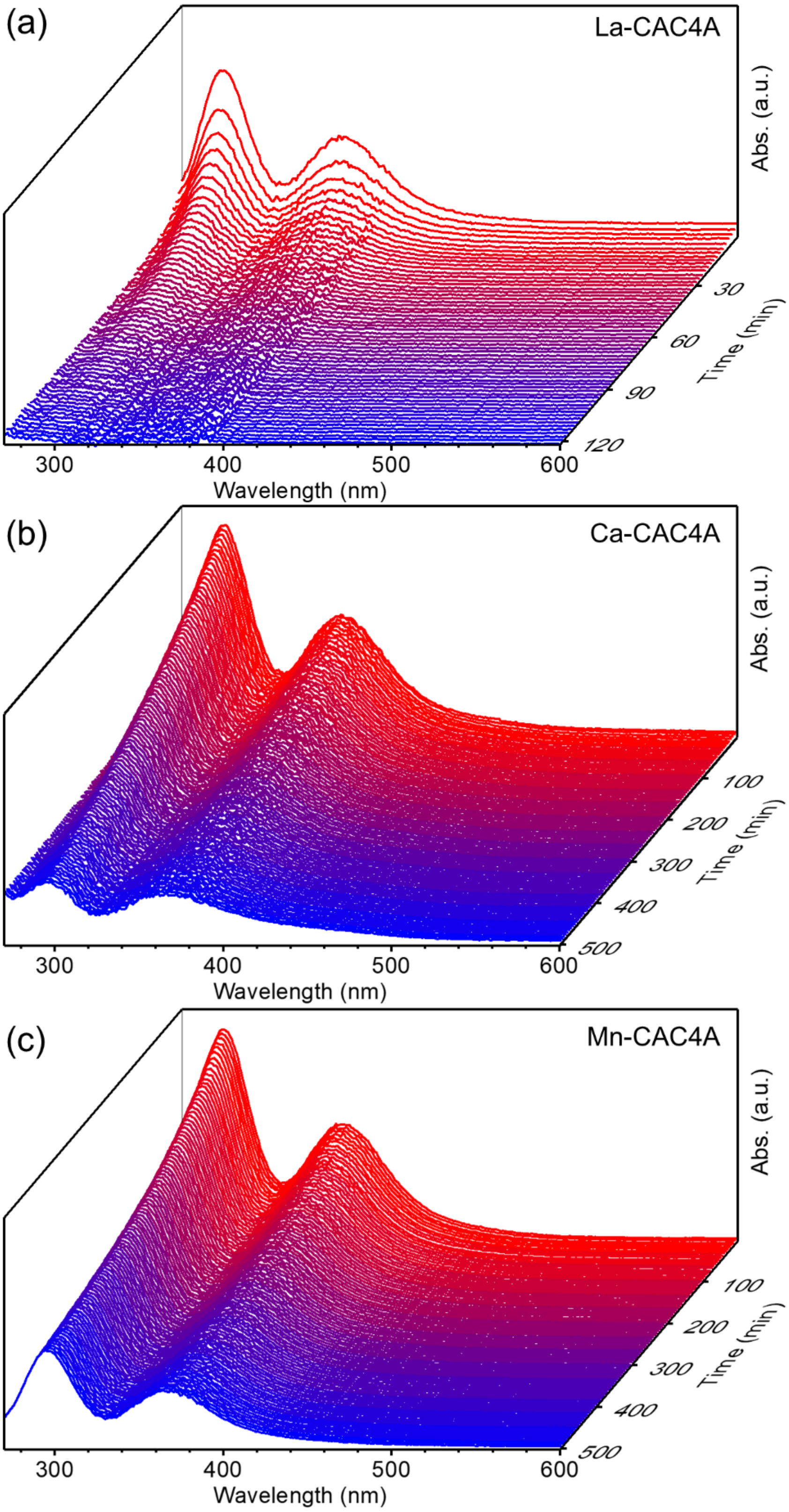

Figure S6. UV-vis spectra of I_2 in DMF with the additions of La-CAC4A (a), Ca-CAC4A (b), and Mn-CAC4A (c).

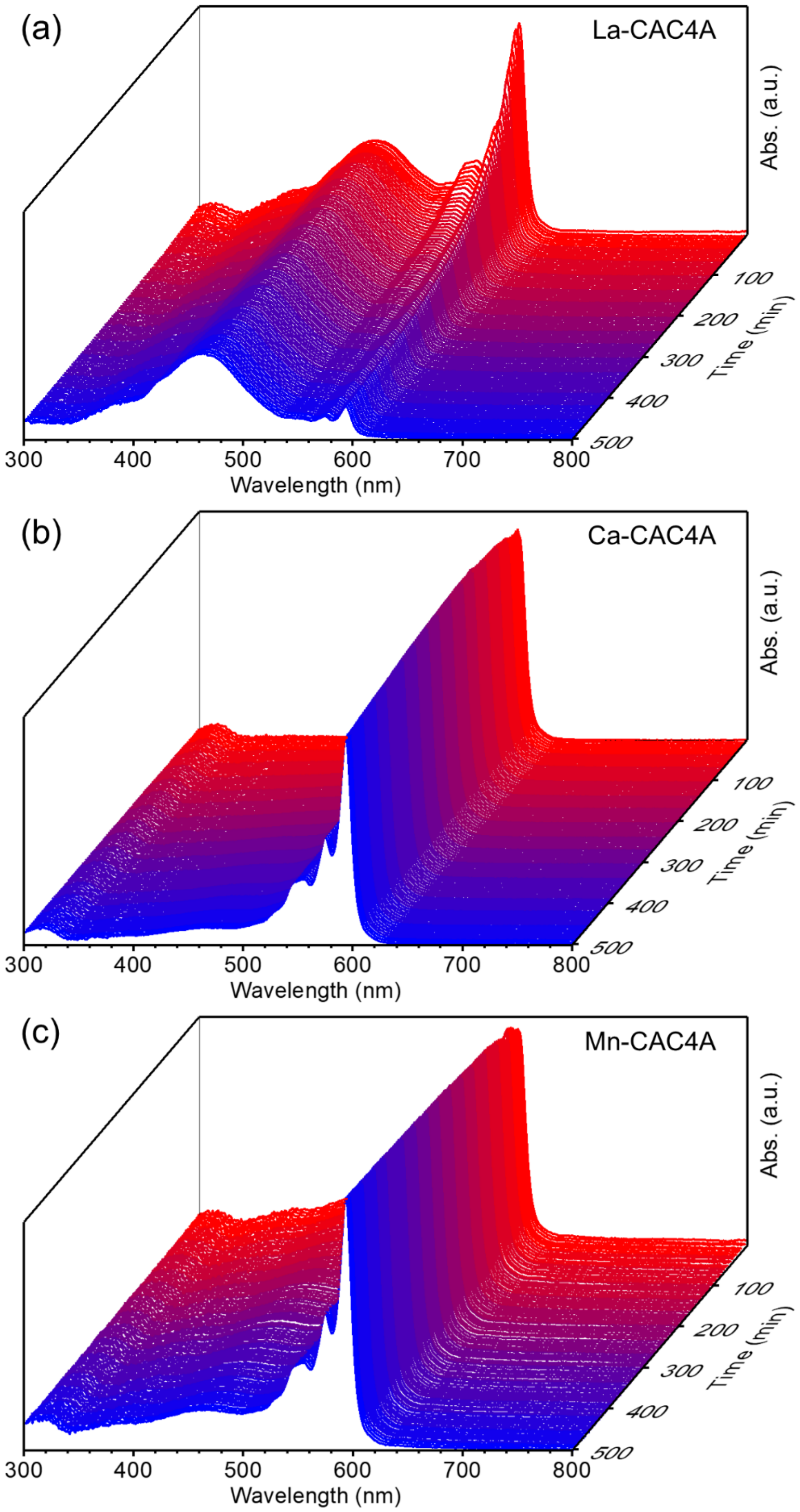

Figure S7. UV-vis spectra of resorufin in DMF with the additions of La-CAC4A (a), Ca-CAC4A (b), and Mn-CAC4A (c).

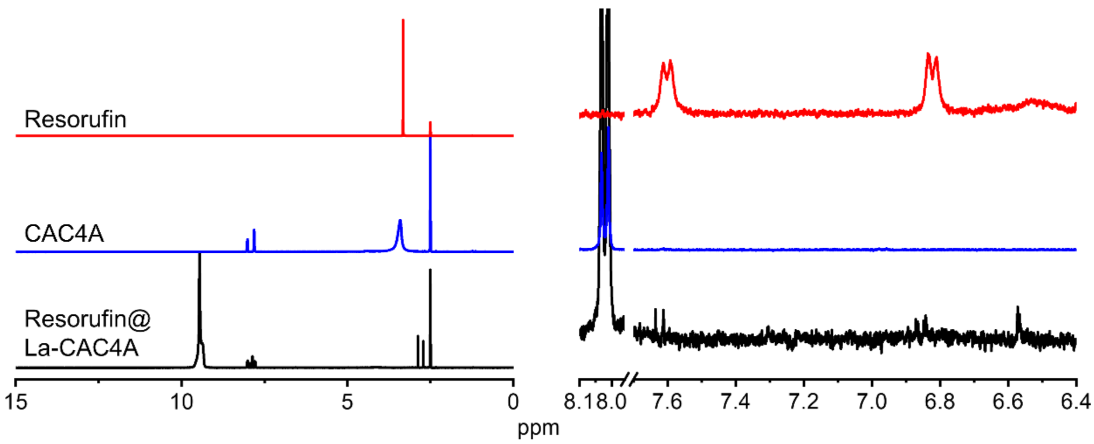

Figure S8. ^{1}H NMR spectra of resorufin, CAC4A, and resorufin@La-CAC4A.

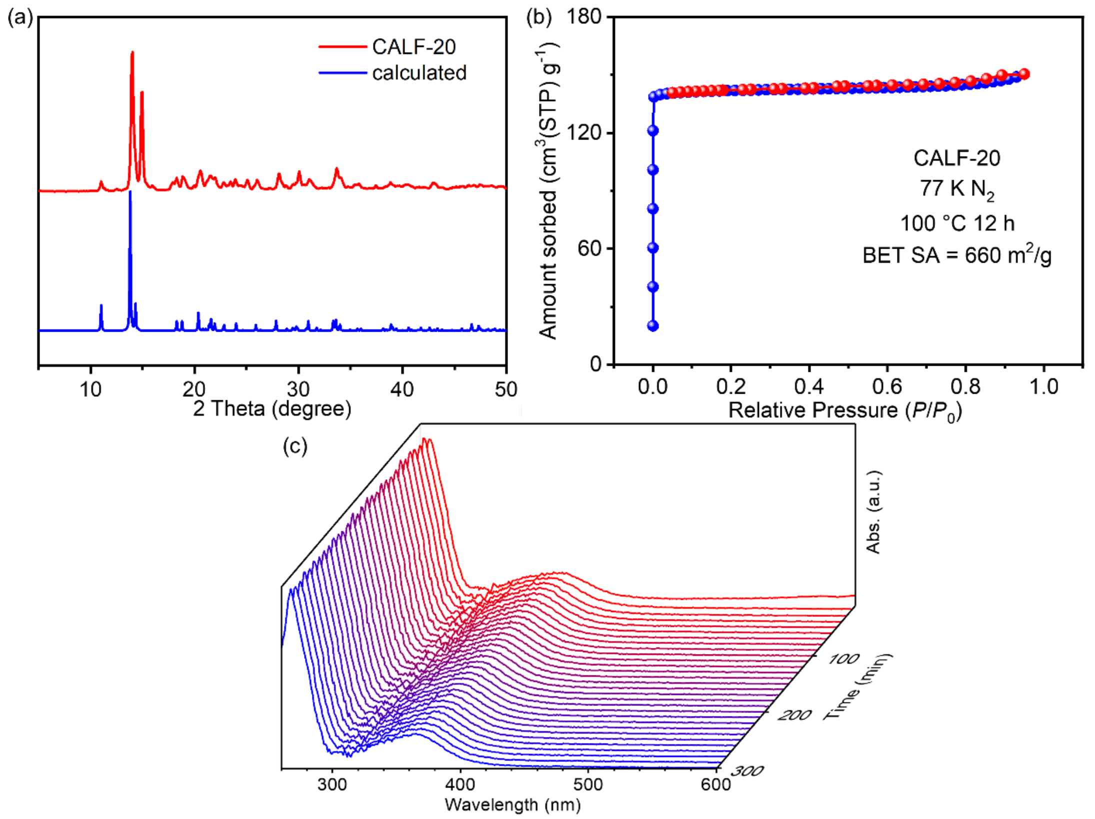

Figure S9. PXRD (a), gas adsorption (b), and UV-vis spectra of I_2 in DMF with the additions (c) of CALF-20.

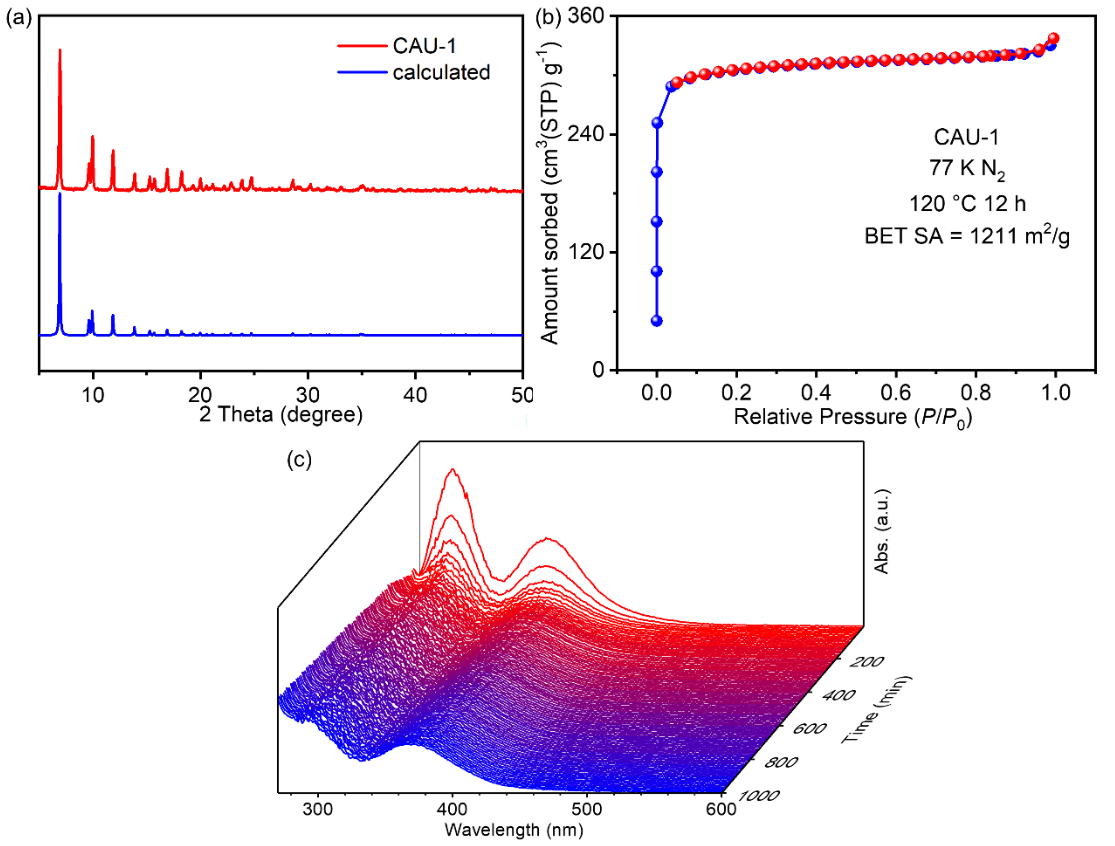

Figure S10. PXRD (a), gas adsorption (b), and UV-vis spectra of I_2 in DMF with the additions (c) of CAU-1.

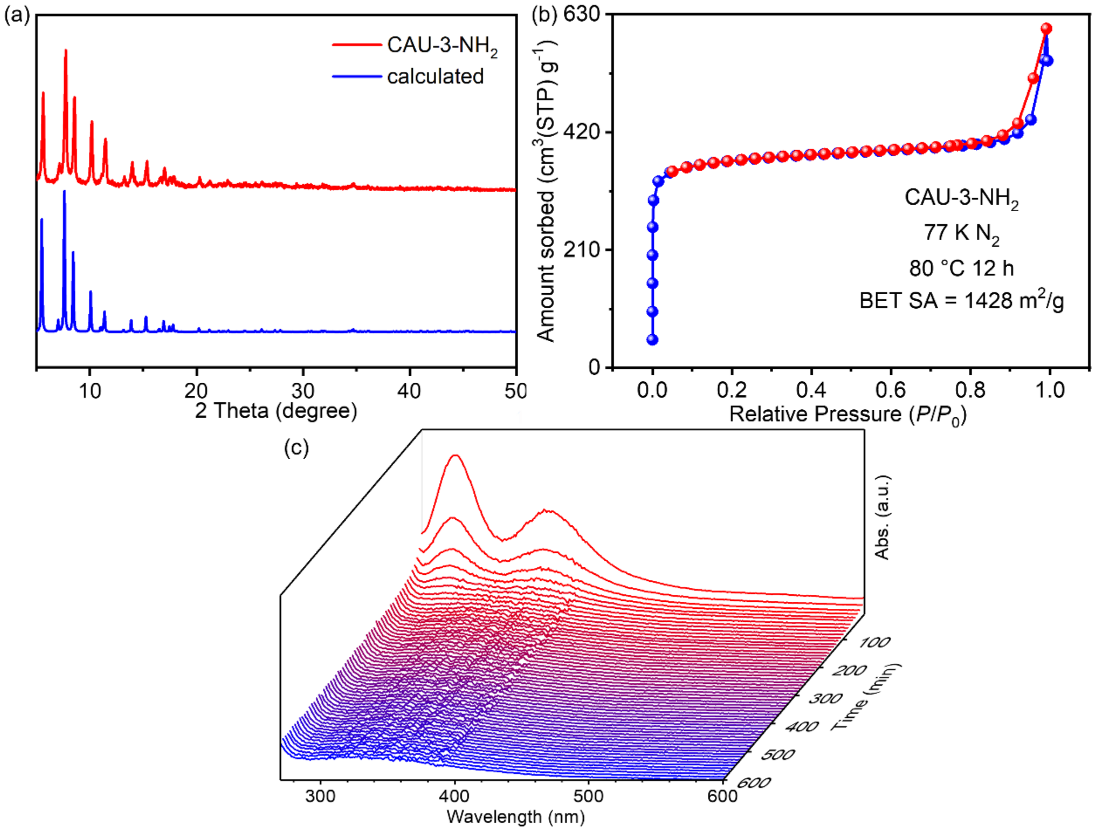

Figure S11. PXRD (a), gas adsorption (b), and UV-vis spectra of I_2 in DMF with the additions (c) of CAU-3-NH_2.

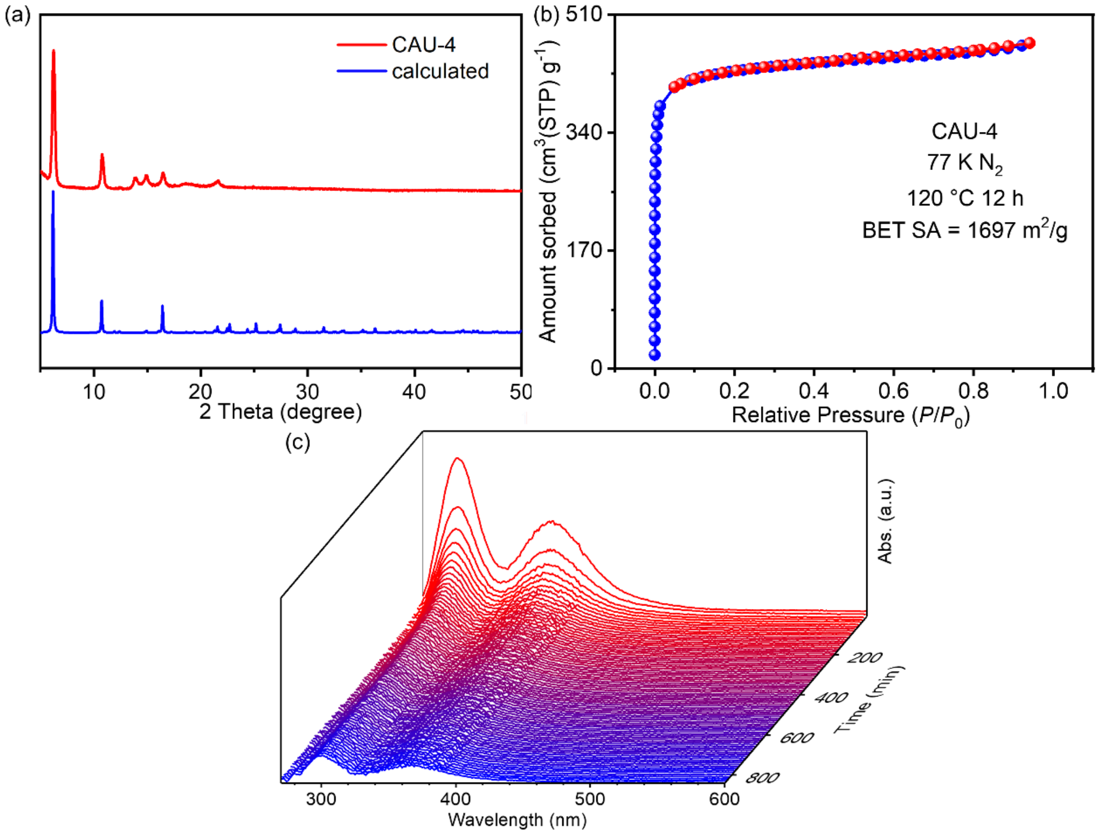

Figure S12. PXRD (a), gas adsorption (b), and UV-vis spectra of I_2 in DMF with the additions (c) of CAU-4.

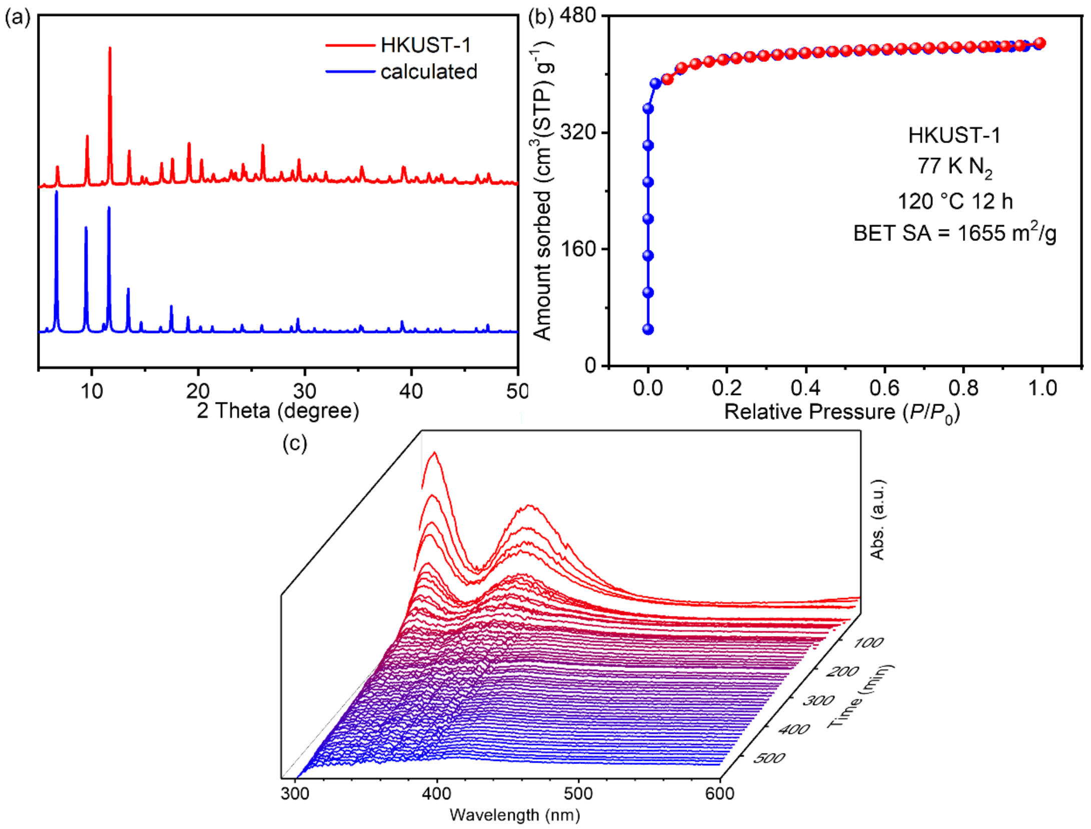

Figure S13. PXRD (a), gas adsorption (b), and UV-vis spectra of I_2 in DMF with the additions (c) of HKUST-1.

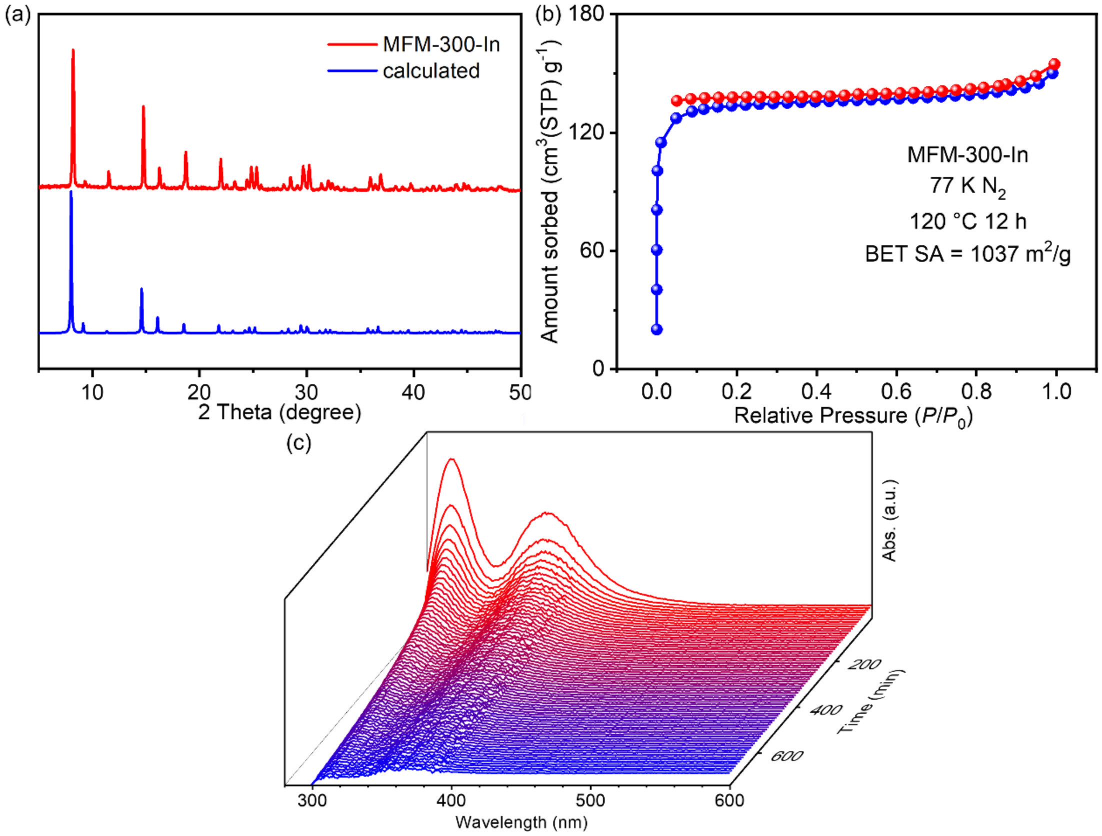

Figure S14. PXRD (a), gas adsorption (b), and UV-vis spectra of I_2 in DMF with the additions (c) of MFM-300-In.

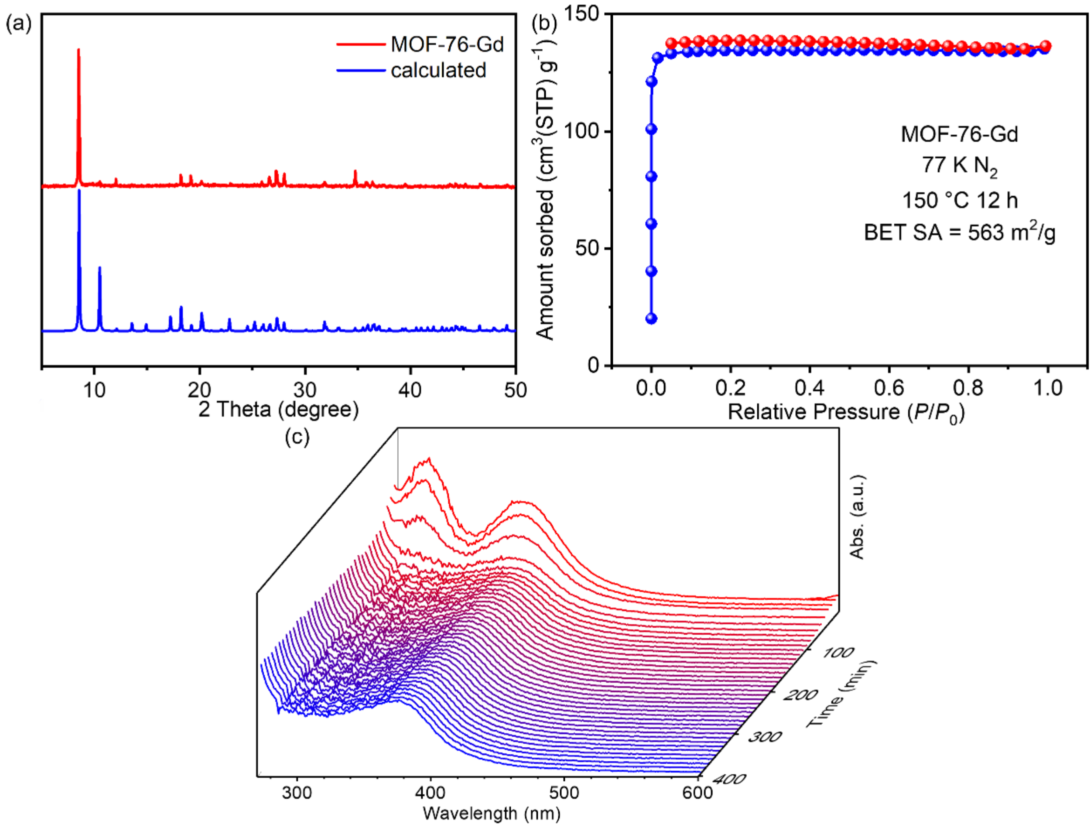

Figure S15. PXRD (a), gas adsorption (b), and UV-vis spectra of I_2 in DMF with the additions (c) of MOF-76-Gd.

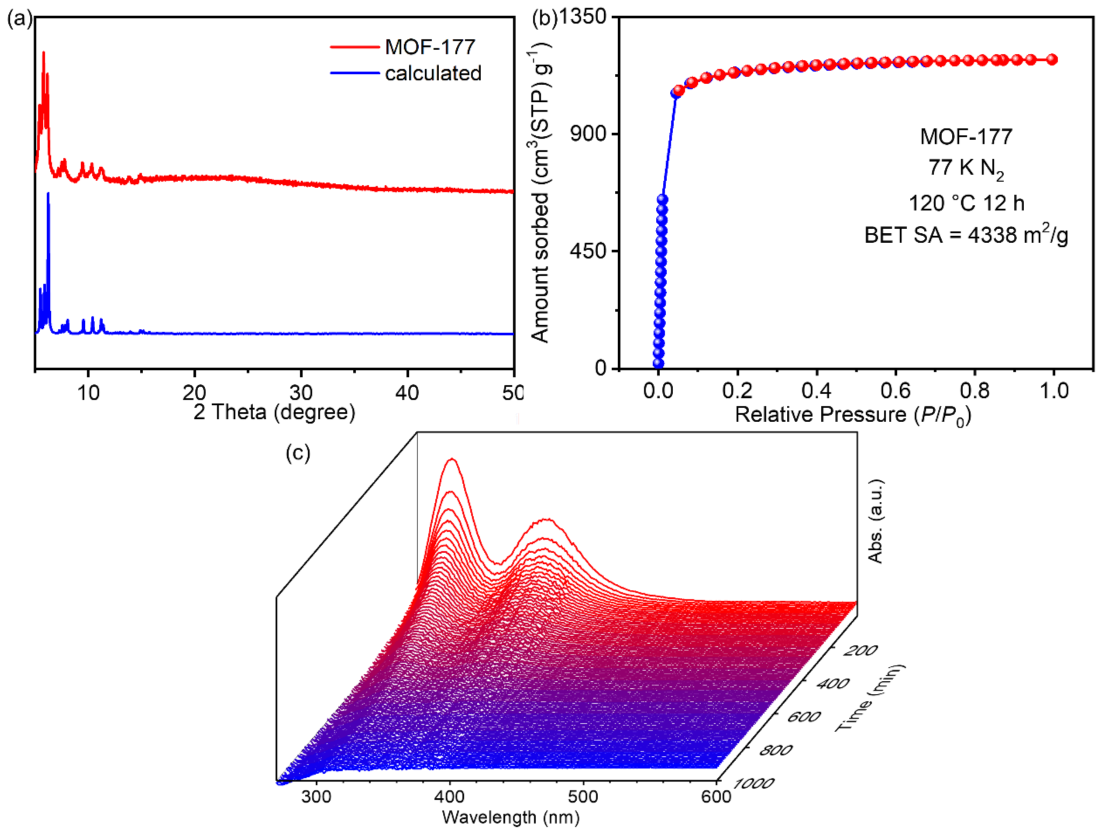

Figure S16. PXRD (a), gas adsorption (b), and UV-vis spectra of I_2 in DMF with the additions (c) of MOF-177.

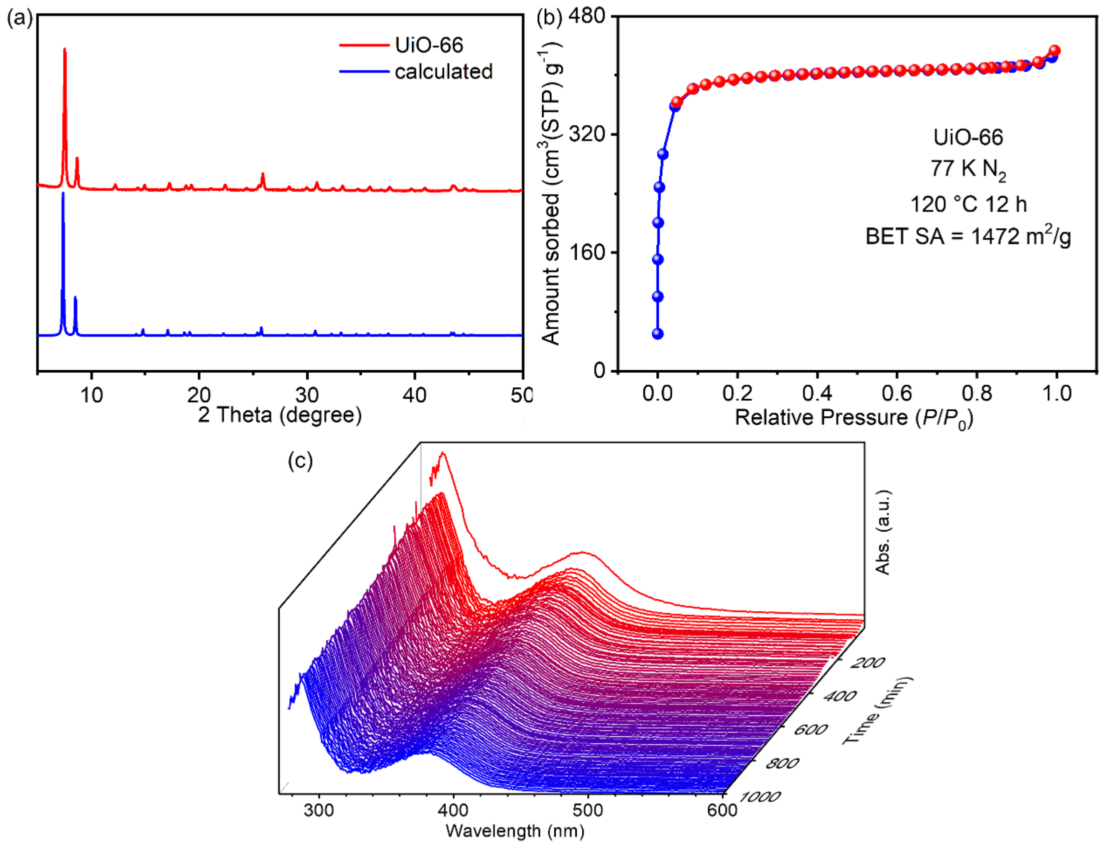

Figure S17. PXRD (a), gas adsorption (b), and UV-vis spectra of I_2 in DMF with the additions (c) of UiO-66.

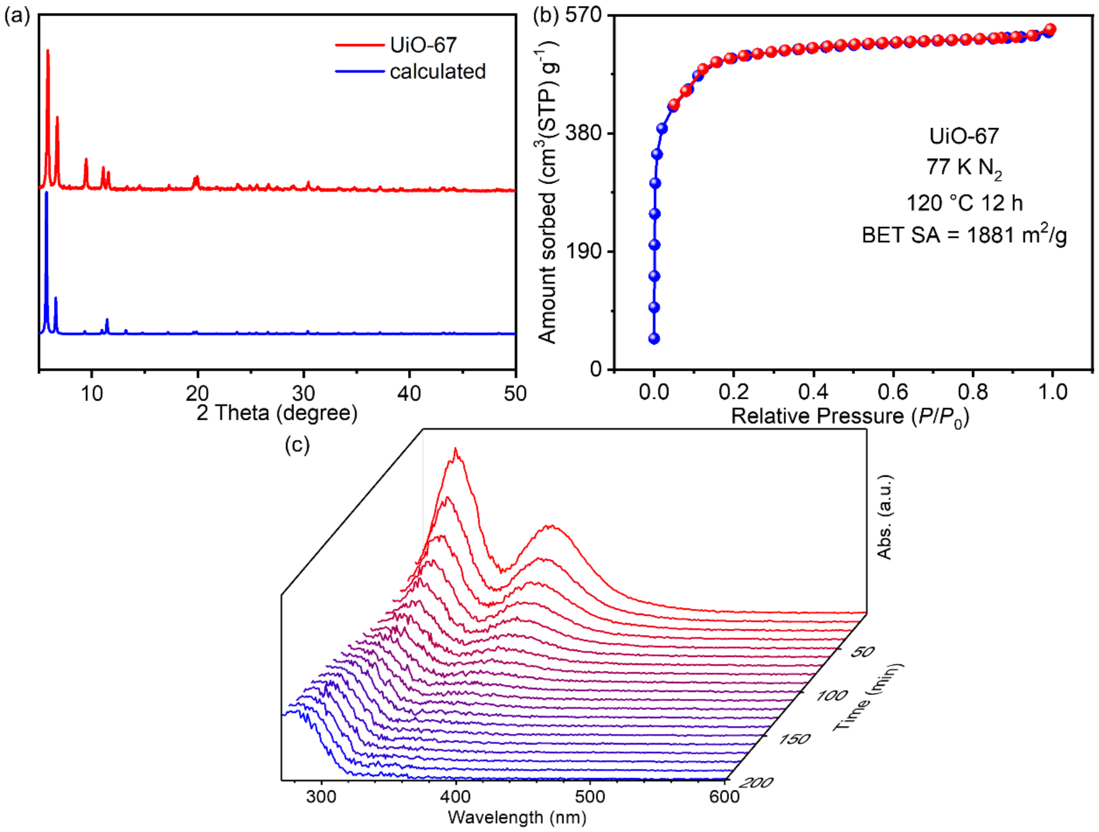

Figure S18. PXRD (a), gas adsorption (b), and UV-vis spectra of I_2 in DMF with the additions (c) of UiO-67.

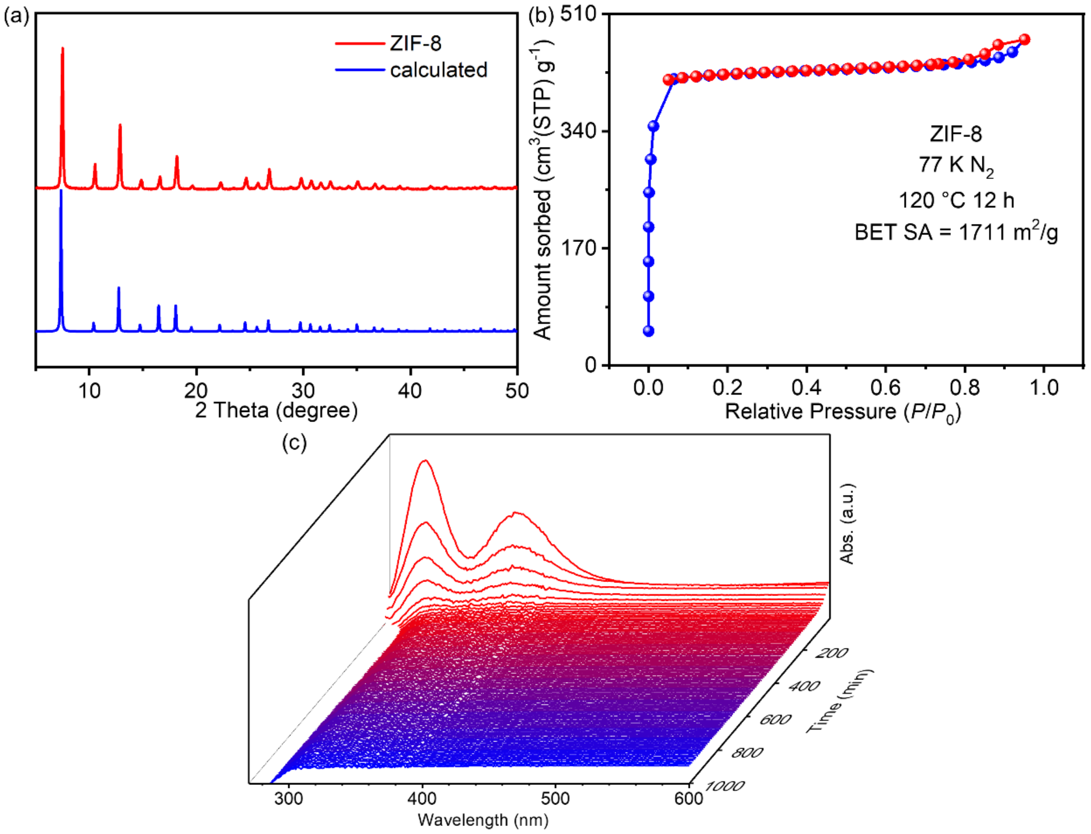

Figure S19. PXRD (a), gas adsorption (b), and UV-vis spectra of I_2 in DMF with the additions (c) of ZIF-8.

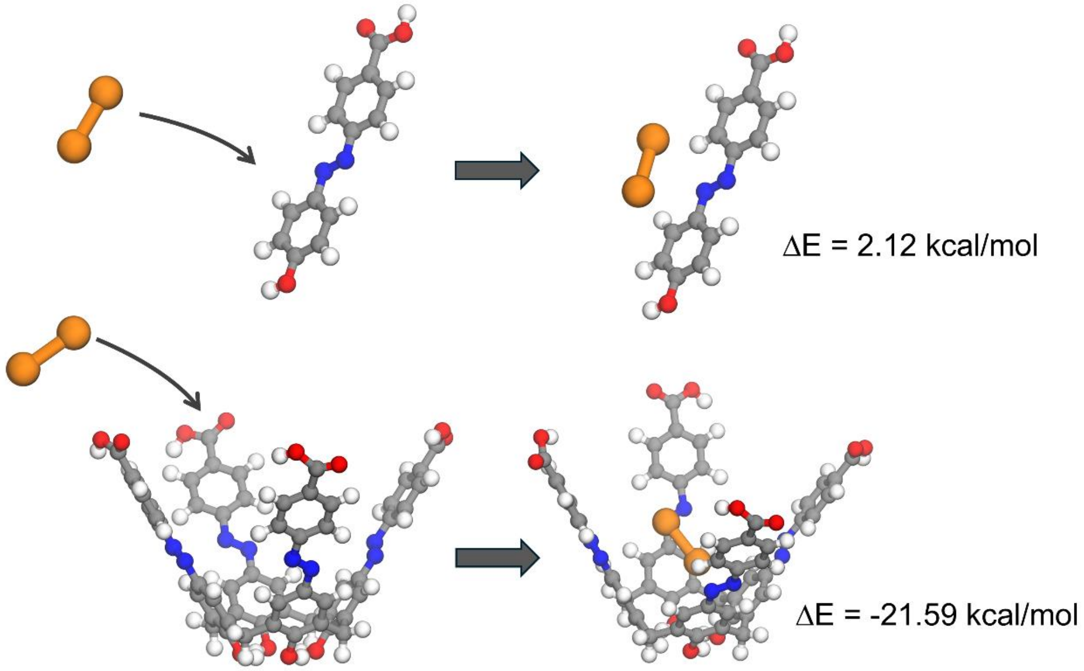

Figure S20. DFT calculation results of the binding energies in the azobenzene monomer and the CAC4A.

Tables

Table S1. Crystallographic data and structure refinement details.

	**La-CAC4A**	**Mn-CAC4A**	**Ca-CAC4A**
CCDC	2359173	2359174	2359171
Formula	$C_{118}H_{86}La_4N_{18}O_{37}$	$C_{75}H_{79}Mn_3N_{14}O_{20}$	$C_{124}H_{100}Ca_5N_{20}O_{37}$
Formula wt	2903.68	1661.34	2662.63
Temperature (K)	100	100	100
Crystal System	triclinic	triclinic	triclinic
Space Group	P-1	P-1	P-1
a (Å)	20.978(4)	10.7344(4)	14.2541(18)
b (Å)	20.983(3)	21.3947(9)	23.356(3)
c (Å)	21.256(3)	21.6429(9)	29.915(3)
α (deg)	72.953(13)	70.911(4)	79.060(8)
β (deg)	63.258(16)	86.518(3)	86.249(9)
γ (deg)	80.469(14)	76.627(3)	72.860(10)
Z	1	2	2
V (Å^3)	7982(3)	4569.2(3)	9343.3(18)
ρ_{calc} (g cm^{-3})	0.604	1.208	0.946
μ (mm^{-1})	0.558	3.900	1.764
F (000)	1444.0	1724.0	2760.0
R_{int}	0.1595	0.0905	0.0988
$^aR_1$, $^bwR_2$ ($I > 2\sigma(I)$)	0.1385, 0.3728	0.0618, 0.1415	0.1148, 0.3152
$^aR_1$, $^bwR_2$ (all data)	0.1677, 0.4019	0.1046, 0.1584	0.1516, 0.3510

$^aR_1 = \Sigma||F_o| - |F_c||/\Sigma|F_o|$, $^bwR_2 = [\Sigma w(F_o^2 - F_c^2)^2/\Sigma w(F_o^2)^2]^{1/2}$

	I_2@La-CAC4A	I_2@Mn-CAC4A	I_2@Ca-CAC4A
CCDC	2359169	2359170	2359172
Formula	$C_{118}I_{1.6}La_4N_{18}O_{37}$	$C_{150}H_{139}I_{1.6}Mn_6N_{28}O_{40}$	$C_{124}H_{96}Ca_5I_{0.8}N_{20}O_{37}$
Formula wt	3020.04	3506.56	2760.12
Temperature (K)	100	100	100
Crystal System	triclinic	triclinic	triclinic
Space Group	*P*-1	*P*-1	*P*-1
a (Å)	20.906(4)	10.7609(9)	14.9340(18)
b (Å)	20.962(4)	21.134(2)	23.116(2)
c (Å)	21.253(4)	21.861(3)	30.875(3)
α (deg)	73.672(17)	71.044(12)	79.417(8)
β (deg)	62.346(20)	86.900(9)	79.742(9)
γ (deg)	82.675(16)	76.549(8)	73.327(10)
Z	1	1	2
V (Å^3)	7917(3)	4571.8(10)	9947(3)
ρ_{calc} (g cm^{-3})	0.633	1.274	0.922
μ (mm^{-1})	5.599	6.022	2.633
F (000)	1443.0	1790.0	2837.0
R_{int}	0.1220	0.0785	0.0992
$^aR_1$, $^bwR_2$ ($I > 2\sigma(I)$)	0.1350, 0.3422	0.1333, 0.3295	0.1204, 0.3184
$^aR_1$, $^bwR_2$ (all data)	0.1980, 0.4039	0.1493, 0.3416	0.1618, 0.3584

$^aR_1 = \Sigma||F_o| - |F_c||/\Sigma|F_o|$, $^bwR_2 = [\Sigma w(F_o^2 - F_c^2)^2/\Sigma w(F_o^2)^2]^{1/2}$

References

(1) Sheldrick, G. M. Crystal structure refinement with SHELXL. *Acta Crystallogr., Sect. C: Struct. Chem.* **2015**, *71*, 3-8.

(2) Sheldrick, G. M. A short history of SHELX. *Acta Crystallogr., Sect. A: Found. Crystallogr.* **2008**, *64*, 112-122.

(3) Lin, J.-B.; Nguyen, T. T. T.; Vaidhyanathan, R.; Burner, J.; Taylor, J. M.; Durekova, H.; Akhtar, F.; Mah, R. K.; Ghaffari-Nik, O.; Marx, S.; Fylstra, N.; Iremonger, S. S.; Dawson, K. W.; Sarkar, P.; Hovington, P.; Rajendran, A.; Woo, T. K.; Shimizu, G. K. H. A scalable metal-organic framework as a durable physisorbent for carbon dioxide capture. *Science* **2021**, *374*, 1464-1469.

(4) Jiao, C.; Majeed, Z.; Wang, G.-H.; Jiang, H. A nanosized metal-organic framework confined inside a functionalized mesoporous polymer: an efficient CO_2 adsorbent with metal defects. *J. Mater. Chem. A* **2018**, *6*, 17220-17226.

(5) Reinsch, H.; Feyand, M.; Ahnfeldt, T.; Stock, N. CAU-3: A new family of porous MOFs with a novel Al-based brick: $[Al_2(OCH_3)_4(O_2C\text{-}X\text{-}CO_2)]$ (X = aryl). *Dalton Trans*. **2012**, *41*, 4164-4171.

(6) Reinsch, H.; Krüger, M.; Wack, J.; Senker, J.; Salles, F.; Maurin, G.; Stock, N. A new aluminium-based microporous metal-organic framework: Al(BTB) (BTB = 1,3,5-benzenetrisbenzoate). *Micropor. Mesopor. Mat.* **2012**, *157*, 50-55.

(7) Chui, S. S.-Y.; Lo, S. M.-F.; Charmant, J. P. H.; Orpen, A. G.; Williams, I. D. *Science* **1999**, *283*, 1148-1150.

(8) Zhang, X.; da Silva, I.; Godfrey, H. G. W.; Callear, S. K.; Sapchenko, S. A.; Cheng, Y.; Vitórica-Yrezábal, I.; Frogley, M. D.; Cinque, G.; Tang, C. C.; Giacobbe, C.; Dejoie, C.; Rudić, S.; Ramirez-Cuesta, A. J.; Denecke, M. A.; Yang, S.; Schröder, M. Confinement of iodine molecules into triple-helical chains within robust metal-organic frameworks. *J. Am. Chem. Soc.* **2017**, *139*, 16289-16296.

(9) Capková, D.; Kazda, T.; Čech, O.; Király, N.; Zelenka, T.; Čudek, P.; Sharma, A.; Hornebecq, V.; Fedorková, A. S.; Almáši, M. Influence of metal-organic framework MOF-76(Gd) activation/carbonization on the cycle performance stability in Li-S

battery. *J. Energy Storage* **2022**, *51*, 104419.

(10) Chae, H. K.; Siberio-Perez, D. Y.; Kim, J.; Go, Y.; Eddaoudi, M.; Matzger, A. J.; O'Keeffe, M.; Yaghi, O. M. A route to high surface area, porosity and inclusion of large molecules in crystals. *Nature* **2004**, *427*, 523-527.

(11) Cavka, J. H.; Jakobsen, S.; Olsbye, U.; Guillou, N.; Lamberti, C.; Bordiga, S.; Lillerud, K. P. A new zirconium inorganic building brick forming metal organic frameworks with exceptional stability. *J. Am. Chem. Soc.* **2008**, *130*, 13850-13851.

(12) Park, K. S.; Ni, Z.; Côté, A. P.; Choi, J. Y.; Huang, R.; Uribe-Romo, F. J.; Chae, H. K.; O'Keeffe, M.; Yaghi, O. M. Exceptional chemical and thermal stability of zeolitic imidazolate frameworks. *Proc. Natl. Acad. Sci. U.S.A.* **2006**, *103*, 10186-10191.

(13) TURBOMOLE V7.5 2021, a development of University of Karlsruhe and Forschungszentrum Karlsruhe GmbH, 1989–2007, TURBOMOLE GmbH, since 2007.

(14) Grimme, S. Semiempirical hybrid density functional with perturbative second-order correlation. *J. Chem. Phys.* **2006**, *124*, 034108.

(15) Grimme, S.; Ehrlich, S. Effect of the damping function in dispersion corrected density functional theory. Goerigk, L. *J. Comput. Chem.* **2011**, *32*, 1456-1465.

(16) Weigend, F.; Ahlrichs, R. Balanced basis sets of split valence, triple zeta valence and quadruple zeta valence quality for H to Rn: Design and assessment of accuracy. *Phys. Chem. Chem. Phys.* **2005**, *7*, 3297-3305.
